# Rise and Progress of Clinical Teaching

**Published:** 1861-12

**Authors:** J. L. Ludlow

**Affiliations:** Philadelphia, at the opening of a new Clinical Lecture Room


### RISE AND PROGRESS OF CLINICAL TEACHING.

*Extract from an Address by*

J. L. LUDLOW, M. D.,

*Philadelphia, at the opening of a new Clinical Lecture Room.*

When ignorance and superstition ruled the minds of men, and credulity and fable were the characteristics of the age, we cannot suppose that our science would have shared more than others in enlightenment and true philosophy.

No—though disease stalked abroad, and man saw his fellow man *writhing* under the torture of pain, or parched by feverish heat, or covered with the pestilential leprosy—though the the sightless eye-ball rolled in its socket, as if looking for some one to destroy the cataract which obscured the vision, or some hideous deformity was intruded upon the passer by—yet man, selfish *though he is*, thought little how he might alleviate the sufferings of his race, by examining the *structure* of the human frame and the uses of its parts.

*We*, at this day, wonder why, instead of placing their sick in temples and public places, to beg of the traveller a remedy for diseases and deformities, they should not have endeavored to so investigate the nature of maladies, and so apply remedies as to relieve, if not to cure.

Buildings for the purpose of worship were the principal depots for the afflicted. In the temples of Esculapius, however, we find more particular attention paid to the ills of the race. The priests, it is true, dealt more in mysteries and in charms than in clinical instruction ; but as time elapsed, by the observations made constantly upon the diseased who frequented the temples, something like a science was commenced.

From the Asclepiades, from the master to his scholars, from father to son, through a long line of descendants, the observations in these temples were transmitted, until we find them culminating, by intellectual and hereditary right, in the sage of *Cos—the immortal Hippocrates—the Father of Medicine.*

To *Hippocrates* we are indebted for those recondite and choice observations in Medicine, gleaned and garnered by study *at the bedside*, which has crowned him with an immortal wreath, and transmitted his name and works, to this late day, as wonders of research and philosophy.

Had the precepts of Hippocrates been followed, and observation at the bedside been made the basis of practical medicine, instead of vagaries, loose speculations and hypotheses, the science of Medicine would, no doubt, have attained greater perfection even at that distant period.

But the minds of those who followed the Father of Medicine appeared to loathe the only true method of instruction in any science. Instead of induction guiding their judgments, they appear to revel in a love of crude thoughts, upon which they attempted to build systems upon which they might wrangle and wrestle, if for nothing else than to prove their great powers of mystifying and *darkening*, by words without wisdom, the simplest truths in nature. As man is alike in all ages of the world, they no doubt were very like some of our modern lecturers and speech-makers, who deal much in what the Latin poet calls " *Vox et preterea nihil* "—a fine speech without matter.

With all due deference to the hoary-headed sires of those ages, I must confess that while I wonder at the industry which amassed their huge tomes, I am amazed that they could write so much and do so little.

As we descend the stream of time, passing, as we necessarily must, many important names and eras, we are arrested by the name and fame of *Galen*. *Not*, however, that he did anything for the advancement of Clinical Medicine, but that he, by the food which he administered by his writings, disquisitions, text-books, rather fostered the spirit of controversy which impeded the progress of true observation into the causes of disease for many ages.

If clinical instruction at this time was followed at all, it could only have been by a few in private practice.

It has been asserted by historians, that hospitals do not date earlier than the close of the third or beginning of the fourth century. Upon this, however, there remains a doubt. At any rate, cotemporary with the creation of hospitals, we must notice the celebrated schools of Alexandria, and that of Dschondisabour, in Persia. These schools being in close proximity to the hospitals, the inference is not too strong that they were made use of as a part of medical instruction.

Among the earlier hospital schools, that of Alexandria, in Egypt, was so celebrated, that it is said an assiduous attention upon it conferred the right of practicing medicine.

Previously to this period, hospitals had been more particularly appropriated to the care of the sick; but from this time they gradually fulfilled a two-fold purpose, *that before mentioned*, and also as schools of medical observation and instruction. When the School of Bagdad was founded by the command of Almanzor, it was filled with superintendents from Dschondizabour, where an infirmary had been founded, superior even to that of Alexandria.

As the Arabs proceeded in their conquests, we find hospitals and medical schools following in their wake. Under the protection of that race, (the Saracen Moors, who overrun Spain,) universities and hospitals sprung up in the cities of Seville, Toledo and Cordova. At such hospitals, Ali-Abbas, a noted Arabian, advised, as a great duty, the attendance upon the *Hospital Schools*, there to observe how little the real appearance and characteristics of disease coincided with the description in the books of the times. Although at this time the advantages from Hospital Schools appeared to offer every opportunity for the only proper advance in medical science—namely, by observation, close and critical, at the bedside—nevertheless, it really appears as if they wished to blot out the sun, and substitute the taper for a luminary, in advancing true medicine. Fruitless and vapid discussions assumed the place of strict observation and induction, and disgust drove several to travelling in Italy and France, whence the tide of literature had been flowing. There too, however, the love of writing and declaiming on medicine, instead of observation, existed. But the number of hospitals increased, and we would have supposed that these would have stimulated proper medical culture. *Not so, however.* Writing and speculation, with a mass of crude controversy, still clouded the minds of the Doctors of those times. Revelling in their favorite schemes and dogmas, they almost extinguished the little light which had dawned in the medical world. They most truly forgot the motto of the illustrious Hippocrates—"*Ars longa—vita brevis est.*"

In proceeding, gentlemen, I think it is not inappropriate to the present occasion, to stop a few moments and pay our tribute of veneration and gratitude to two immortal names, Sir Francis Bacon and Thomas Sydenham. The former the father of Inductive Philosophy; the latter the restorer of Hippocratic Medicine. The former the pioneer in correct reasoning; the latter the applier of that system to the development of our science. The influence of the Baconian philosophy upon Sydenham we may learn from the following passages:

"As truly as the physician may collect points of diagnosis from the minutest circumstances of the disease, so truly may he also elicit indications in the way of therapeutics. So much does this statement hold good, that I have often thought that, provided with a thorough insight into the history of any disease whatsoever, I could invariably apply an equivalent remedy—a clear path being thus marked for me, by the different phenomena of the complaint. These phenomena, if carefully collated with each other, lead, as it were, by the hand, to those palpable indications of treatment which are drawn, not from the hallucinations of our fancy, but from the innermost penetration of nature.

"By this ladder, and by this scaffold, did Hippocrates ascend the lofty sphere. The Romulus of Medicine, whose heaven was the empyrean of his art, he it is whom we can never duly praise. Herein consisted the theory of the divine old man. It exhibited the legitimate operations of nature, put forth in the diseases of humanity. The vain efforts of a wild fancy, the dreams of a sick man, it did *not* exhibit."

I could quote further, with advantage to us all, but I must hasten onward.

Passing now to the middle of the seventeenth century, our attention is arrested by the establishment of *Cliniques* in Holland—that land more maligned and calumniated than any country in Europe, and yet a land around which more glorious recollections cluster than any land in the Eastern hemisphere; a spot which suffered more for civil and religious liberty than almost any part of the world—the country of William and Mary. The country which gave William and Mary to England, and by them the Toleration Act. The home of the oppressed, the refuge of the Puritans before they set sail across the broad Atlantic, for New England's rocky coast. The land which, when given a choice between money and a *University*, chose a University. A land of literature and letters, of science and art. A people who rescued their country from the sea. A land of dykes and canals, and a people who, when they had not land enough, pumped out a lake. And yet they call this people, with a sneer, phlegmatic Dutchmen. I cannot tell whence came the delusion, excepting that they, with all their industry, and prowess, and literature, knew how to enjoy life. The old men with their long pipes, and the venerable dames with their huge caps and their well stored larders, which they knew how to prepare; with easy consciences, and a sufficient rotundity of body to keep them warm in winter, and perhaps a good glass of Holland gin for a night-cap. If you doubt what I have said, read Schiller, and Davies, and Motley, and Brodhead, and Prescott, and I know you will bear me out in my assertions. But to return.

In 1643, clinical establishments flourished in Holland. The most distinguished was that at Utrecht, under *Strœten*, and at Leyden, under *Huernius*. He was the first to teach bedside medicine upon a comprehensive scale. After examining closely the patient in presence of his pupils, he expatiated upon the nature of the disease, and also frequently caused post-mortem examinations to be made.

In addition to Huernius, we have De Laboe, who was the first *to publish* a connected series of bedside observations with remarks, and this system was pursued by his successors. To De Laboe, of Leyden, we are indebted for the first *Clinical Reports*. The next great clinical teacher is the illustrious Boerhave. Under his instruction some of the most distinguished physicians of Europe were educated, who scattered throughout the land the fruits of the good seed which had been sown by Boerhave at Leyden.

Now we find the spirit of proper medical instruction, based upon Clinical investigation, spreading to Scotland, and afterwards to Vienna, whose schools soon eclipsed the mother institution.

In Scotland, Dr. Rutherford, in 1748, commenced his course of Cliniques in the Royal Infirmary in Edinburgh, in which he was soon joined by other professors in 1757. I have in vain endeavored to ascertain when Cliniques were introduced into England and Ireland, where we know they at present exist in great perfection. I gather, however, from the histories of some of the older hospitals, that the Hospital of *St. Bartholemew*, was instituted in connection with the priory of St. Bartholemew in 1122, and after undergoing various modifications in its charter, in which Christ's Hospital, Bridewell, and St. Thomas were included, it became more perfectly established in 1752. Yet, from an historical oration, delivered at the opening of one of her sessions of lectures, I find that it was not before 1822 that John Albernethy founded the *present system* of lecturing, though Percival Pitt delivered occasional lectures some 80 years ago. St. Thomas' Hospital was founded in 1213, and after being subjected to many changes, was finally well endowed in 1782, to which a medical school was attached, but the exact date I cannot give.

Guy's Hospital was founded by Thomas Guy, of London, in 1725, and in 1760 an arrangement was made within St. Thomas' Hospital, by which the privileges of both hospitals were extended to the pupils of each. In Dublin, the Meathe and Sir Patrick Dunn's Hospitals, and others have been open for years for Clinical instruction, but when they were opened I have not been able to ascertain.

Five years after the Cliniques had been originated by Dr. Rutherford, in Edinburgh, a Clinical institution was established at Vienna by Boerhave, where lectures were delivered by *Van Swieten*, who was succeeded by De Haen, who gave the results of his observations and experience to the public, supported at times by necrological examinations in his *Ratio Medendi*, a woık of sixteen small volumes, in which he insisted upon the Hippocratic method of investigating disease. De Haen was followed by Stoll, who gave a still greater impulse to the Viennese Clinique.

While the Clinique was thus progressing in Vienna, we now find Dr. Cullen giving Clinical lectures in the Royal Infirmary of Edinburgh, assisted by Drs. Wyeth and Monro, and afterwards Gregory and Home.

The combined influence of the good effects of Cliniques as subservient to high toned medical instruction, in Holland, Vienna and Scotland, excited at this time, a general interest in Clinical study in other schools of Europe, in Gottingen and Sarthe, &c. In Italy in 1715, a Clinique was established by the Pope, in connection with the hospital at Rome, mid all the pomp and splendor of the Church, in the presence of cardinals and prelates, with orisons and vespers, and the full diapason of the multitude, Lancisci was inaugurated as Chief of the Clinique in the vast Hospital of the Holy Ghost. In passing, I may remark, that we find a species of Clinique attached to the Hospital of St. Francis in Padua, as far back as 1578, but of this we know very little, it probably, from all we can gather in regard to it, did not deserve the name, even in the most restricted sense.

In the University of Pavia, in 1781, Tissot and Scarpa occupied Clinical chairs; while at Bologna, Tommisini was occupied, and at Milan, Rasori.

In Spain, also, we find Cliniques established, and pupils were compelled to see a town practice of two years, before entering upon private duties.

In Russia Clinical instruction has been established since 1765, and continued upon a most extensive and vigorous scale. The more proficient Clinists, to a certain number, are sent at the expense of Government, to other countries, to gather what is new in medical or surgical knowledge. In Prussia, it is said that Cliniques are more insisted upon, than even in Russia.

In the cursory glance which I have given you, gentlemen, of Clinical establishments, I have designedly said nothing of France. That country deserves especial commendation, for since their inception, the attention which has been paid to Clinical instruction, has vastly surpassed, as a whole, that of any other country on the globe. Previous to 1786, when reforms were introduced into the University of Caen, and a chair of practical medicine was attached to the Hotel Dieu, De Bois, physician to La Charité, gave Clinical lectures to a limited number of pupils.

During the convulsions of the state which lacerated France at the close of the 18th century, medicine alone remained stationary. Her votaries, alive to the interests of humanity, were ceaseless in their efforts to advance medical science, and during every lull in the political tempest, the subject was pressed upon the attention of the Government.

In the year 1794, Cliniques became attached directly to the faculties of medicine, and simultaneously the schools were provided with Clinical professors. Desault occupied a chair of Clinical surgery, and at the death of De Bois, Corvisart was appointed physician to La Charité, and in 1795 professor of Clinical medicine.

Corvisart being selected as medical adviser to the Emperor Napoleon, on the first day of the consulate, used that influence with the first consul which his position and talents afforded him, and which might have been expected from one so distinguished in the annals of medicine. Among the pupils of Corvisart we find Bayle Lannec and Baron Dupuytren. Pathology engaged his attention. Percussion, and investigations upon the diseases of the heart were illustrated at the bedside of the patient. During the professorship of Corvisart, many of the most eminent practitioners of succeeding years were educated, and thence dispersed throughout various parts of the empire. At this time more attention was paid to Pathological Anatomy, and post-mortem examinations were extensively and vigorously carried out. In addition to the Clinique in Paris, Fouqué founded Clinical instruction in the old and time honored University of Montpelier, in which he was ably seconded by Delpech. At *Strasburg*, after a great length of time, and much persuasion, an Obstetrical Clinique was founded.

Though France had been late in entering the Clinical field, yet she soon made up by her efficiency, what she had lost by tardiness.

The multitude of her Clinical institutions are unrivalled. There is not a city in the world where equally numerous and varied Clinical courses are delivered as in Paris.

The science of practical medicine owes much to the energy and industry of our Galic brethren. No where has there been so close an analysis of disease, and so scrutinizing investigation.

In later years the discoveries of the teachers of Germany, Austria, England, Ireland, America, have added much to our previous knowledge of Physiology, Pathology, and the general science of medicine. But for a long period before, France must be acknowledged to have done more than any other country, and all this may be attributed to the spirit of research, suggested by bedside observation and Clinical lectures.

